# Consensus on managing open ankle fractures in the frail patient: understanding the pathology, clinical outcomes, and common problems utilizing a modified Delphi process

**DOI:** 10.1302/2633-1462.53.BJO-2023-0155.R1

**Published:** 2024-03-22

**Authors:** Enis Guryel, Jo McEwan, Amir A. Qureshi, Alastair Robertson, Raju Ahluwalia, Open Ankle Fracture of the Frail (OAFF) Collaborative Group

**Affiliations:** 1 University Hospitals Sussex, Brighton, UK; 2 University Hospital Southampton, Southampton, UK; 3 King’s College Hospital, London, UK; 1 University Hospital Southampton NHS Foundation Trust, Southampton, UK; 2 Royal Liverpool University Hospital, Liverpool, UK; 3 South Tees Hospitals NHS Foundation Trust, Middlesbrough, UK; 4 Royal Stoke University Hospital, Stoke-on-Trent, UK; 5 St. George’s Hospital, London, UK; 6 Sheffield Teaching Hospitals, Sheffield, UK; 7 Oxford University Hospitals, Oxford, UK; 8 King's College Hospital, London, UK

**Keywords:** frailty, open fracture, elderly fracture, ankle fracture, management, ankle fractures, open fractures, orthopaedic injuries, fragility fractures, plastic surgeons, hip fracture, debridement, lower limb, Limb Reconstruction

## Abstract

**Aims:**

Ankle fractures are common injuries and the third most common fragility fracture. In all, 40% of ankle fractures in the frail are open and represent a complex clinical scenario, with morbidity and mortality rates similar to hip fracture patients. They have a higher risk of complications, such as wound infections, malunion, hospital-acquired infections, pressure sores, veno-thromboembolic events, and significant sarcopaenia from prolonged bed rest.

**Methods:**

A modified Delphi method was used and a group of experts with a vested interest in best practice were invited from the British Foot and Ankle Society (BOFAS), British Orthopaedic Association (BOA), Orthopaedic Trauma Society (OTS), British Association of Plastic & Reconstructive Surgeons (BAPRAS), British Geriatric Society (BGS), and the British Limb Reconstruction Society (BLRS).

**Results:**

In the first stage, there were 36 respondents to the survey, with over 70% stating their unit treats more than 20 such cases per year. There was a 50:50 split regarding if the timing of surgery should be within 36 hours, as per the hip fracture guidelines, or 72 hours, as per the open fracture guidelines. Overall, 75% would attempt primary wound closure and 25% would utilize a local flap. There was no orthopaedic agreement on fixation, and 75% would permit weightbearing immediately. In the second stage, performed at the BLRS meeting, experts discussed the survey results and agreed upon a consensus for the management of open elderly ankle fractures.

**Conclusion:**

A mutually agreed consensus from the expert panel was reached to enable the best practice for the management of patients with frailty with an open ankle fracture: 1) all units managing lower limb fragility fractures should do so through a cohorted multidisciplinary pathway. This pathway should follow the standards laid down in the "care of the older or frail orthopaedic trauma patient" British Orthopaedic Association Standards for Trauma and Orthopaedics (BOAST) guideline. These patients have low bone density, and we should recommend full falls and bone health assessment; 2) all open lower limb fragility fractures should be treated in a single stage within 24 hours of injury if possible; 3) all patients with fragility fractures of the lower limb should be considered for mobilisation on the day following surgery; 4) all patients with lower limb open fragility fractures should be considered for tissue sparing, with judicious debridement as a default; 5) all patients with open lower limb fragility fractures should be managed by a consultant plastic surgeon with primary closure wherever possible; and 6) the method of fixation must allow for immediate unrestricted weightbearing.

Cite this article: *Bone Jt Open* 2024;5(3):236–242.

## Introduction

Ankle fractures are common injuries and approximately 25% occur in patients aged over 60 years. It is the third most common fragility fracture,^1,2^ and 40% of ankle fractures in the frail are open, with an estimated incidence of 1.5% of all ankle fractures.^3^ Open fractures in frail patients represent a complex clinical scenario of pathology and end-stage disease with morbidity and mortality rates similar to hip fracture patients.^4^ The fracture pattern is usually different, and due to the fragility of the skin, the open wound is usually on the medial side with minimal soft-tissue stripping.

As with many orthopaedic injuries, the population sustaining ankle injuries are increasingly older patients with medical comorbidities presenting with fragility fractures.^3^ Closed ankle fractures have a one-year mortality of 12% for patients who require admission to hospital.^5^ Frail patients are at a greater risk of sustaining an open ankle fracture compared to closed fractures,^6^ leading to open injuries in 40% of frail patients with ankle fractures.^7^ For this cohort of patients, one-year mortality rates have been reported up to 30%, which is comparable to the neck of femur fracture population.^4,8^ In the frail patient, the rate of complications after plate fixation of the ankle has been described as between 25% and 40%,^9^ with 30% of frail patients having wound infections.^10^ Compared to fit patients, the frail patient is at risk of increased mortality rates following multiple surgeries,^11^ significant sarcopaenia following just seven days bed rest,^12^ and restricted rehabilitation caused increased bed sores, hospital-acquired infections, and veno-thromboembolic events.^13^

This review and report followed a modified Delphi method involving a panel of experts and delegates to discuss and identify key clinical decisions and organizational problems in the management of open ankle fractures in the frail. Furthermore, it aimed to identify common problems and concerns that exist in the surgical community in managing these patients and potentially reduce the morbidity and mortality.

## Methods

We report a three-step modified Delphi process. In the first step, the senior author (EG) sent a questionnaire to key stakeholders and experts to solicit clinically relevant questions and topics. The survey was sent to each major trauma centre in the UK via email, and there were 36 respondents.

In the second stage, an in-person meeting of a group of experts was undertaken at a specialist session at the British Limb Reconstruction Society (BLRS) meeting in 2022, to discuss the management of the common problem of open ankle fractures in the frail patient. The discussion was led by AT, EG, RA, AR, and IM (see Acknowledgements), and the opinion sought from a panel of experts from different bodies associated in the management of these patients. Representatives with a vested interest in best practice were invited from the British Foot and Ankle Society (BOFAS) (LM), British Orthopaedic Association (BOA) (BH), Orthopaedic Trauma Society (OTS) (WE), British Association of Plastic & Reconstructive Surgeons (BAPRAS (AVG), British Geriatric Society (BGS) (MB), and the BLRS (OM) (see Acknowledgements). All members undertake the management of these complex ankle fractures.

The topics discussed focused on five key areas: 1) current guidelines and best practice; 2) the concept and problems of patients with frailty; 3) soft-tissue wound management (utilizing simple soft-tissue preserving closure in patients with frailty); 4) use of single stage surgery; and 5) unrestricted rehabilitation and early weightbearing

After each topic was discussed, facilitators reviewed and sorted through the answers and key statements were produced. At the end of the process, a further vote based on an agree/disagree question took place to produce a mutually agreed consensus from the expert panel for the management of frail patients with open ankle fractures.

## Results

Of the 36 respondents to the survey, 29 (80%) were orthopaedic surgeons and seven (20%) plastic surgeons, with over 70% stating that their unit treats more than 20 open elderly ankle fractures per year. There was a 50:50 split regarding if the timing of surgery should be within 36 hours, as per hip fracture guidelines, or 72 hours, as per open fracture guidelines. Over 95% of respondents stated that open elderly fracture patients are managed together with plastic surgeons, and 75% would attempt primary wound closure, whereas 25% would utilize a local flap. There was no agreement from the orthopaedic surgeons on the type of fixation method, and 75% stated they would permit weightbearing immediately postoperatively.

In the second stage, during a specialist session at the BLRS meeting in 2022, the results of the survey were discussed with experts from the BOFAS, BOA, OTS, BAPRAS, BGS, and BLRS. The summary of the discussion and views that were presented at the meeting are outlined below:

### Current guidelines and best practice

Several pre-existing guidelines to delineate the management of different components of an open ankle fracture in frail patients which were identified by the panel and discussed: the BOA Standards for Trauma and Orthopaedics (BOAST) open fracture guideline (open fractures 2017);^14^ the BOAST ankle fracture guideline (management of ankle fractures 2016);^15^ and the BOAST care of the older or frail orthopaedic trauma patient guideline (care of the older or frail orthopaedic trauma patient 2019).^16^

All these guidelines focus on aspects of care applicable to this clinical scenario, yet none of them fully addresses the entirety of the problem of open ankle fractures in the elderly frail patient. The common opinion was that this represents a different clinical entity to the high-energy open fracture in a young patient, and it was agreed that the goals are different for this cohort, and therefore the strategy to achieve them will also be different. It was felt that the practice of staging open fractures was not appropriate to frail patients nor necessary to achieve the best outcome given the risks associated with multiple operations, sarcopaenia, bed sores, and hospital-acquired infections.

### The concept and problems of patients with frailty

There are many descriptors used to define the elderly population. Frailty, fragility, and elderly are often used interchangeably; however, the terms are not synonymous, and it is important to clarify the terminology. Frailty is defined as an age-related multidimensional state of decreased physiological reserves that lead to diminished resilience and increased vulnerability to stressors.^17^ It is the common final pathway for physical decline due to environmental and disease-related factors and can be considered as a pre-disabled condition.^18^ Frailty is therefore different to ageing. Frailty is also not solely described through comorbidities, which are disease diagnoses that can be managed.

Within the context of an open ankle fracture, there are numerous stressors that will result in increased morbidity and mortality in an older person with frailty. The frail patient has reduced physiological reserve, which impairs their ability to cope with trauma and surgery.^11^ This is further exacerbated by multiple surgeries, leading to repeated anaesthesia and repeated fasting of a patient. The panel agreed multiple surgeries were felt to increase the duration of bed-bound immobility prior to definitive surgery, resulting in increased risk of hospital-acquired infections, veno-thromboembolic events, pressure sores, and significant sarcopenia after only seven days of bed-bound immobility.^12^ Anaesthetic agents have also been linked to postoperative cognitive decline, and at the extremes of ages and the lasting impact of multiple surgeries within a short time frame on frail patients has not been quantified.^19^

There is good evidence that unrestricted rehabilitation allowing full weightbearing improves outcomes in hip fracture patients. However, there is a reluctance to allow full weightbearing for periarticular fractures, including tibial plateau, plafond, and ankle fractures. There have been efforts to try and form a consensus opinion to create guidelines for weightbearing patients;^13^ however, there are often not implemented in clinical practice.

### Soft-tissue wound management

We strongly agree that the BOAST open fracture guidelines are adhered to in terms of the surgery being performed by a multidisciplinary team involving an orthopaedic and plastic surgeon. As a low-energy injury, the index procedure should take place within 24 hours of the injury. The differences we are examining are in terms of how that care is delivered.

Soft-tissue reconstruction options range from simple closure to free flaps according to the “reconstructive ladder”. Open ankle fractures pose a complex issue due to the subcutaneous nature of the bones and fewer options for local flaps. Although age and comorbidity should not be used as a determinant for offering limb reconstruction with free flaps,^20^ frailty, as previously discussed, is a more relevant factor. Frail patients are less likely to tolerate prolonged surgical time and and are poorly reflected in American Society of Anaesthesiologists grading, which are significant predictors of medical complications postoperatively.^21^

**Fig. 1:**
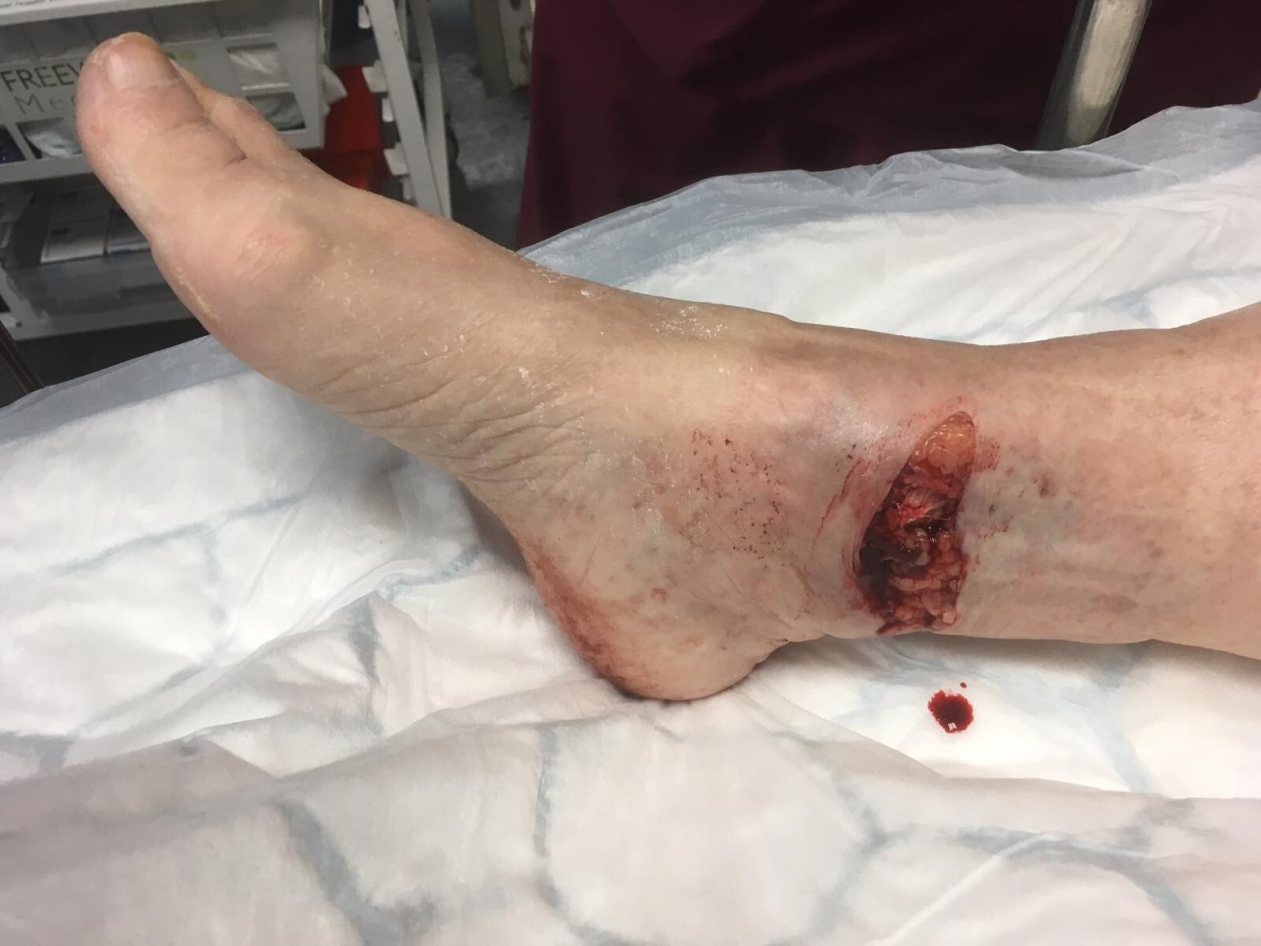
Clinical photograph of a right open ankle fracture, demonstrating a transverse medial wound in which the skin has failed under tension.

In patients with frailty, the panel agreed to advocate a principle of “judicious debridement”, rather than an oncological style resection in the management of the soft-tissue debridement. Particularly in the most common scenario of a transverse medial skin split (Figure 1), over-zealous debridement in frail patients can lead to unnecessary tissue excision in patients who are not good candidates for advanced reconstructive options, such as free flaps. A more pragmatic approach to conserve tissue in frail patients is advised.

The type of osseous stablization used also has an influence on the soft-tissue reconstruction. The optimum management for both the soft-tissue and the bony injury is best made through a combined orthoplastic approach (BOAST open fractures).^14^ We advise that in situations in which the soft tissues would be compromised by a fixation implant, such that a more complex soft-tissue reconstruction would be required, then the orthopaedic strategy should be modified to a more minimally invasive option. For example, if placing a locking plate on the medial side would compromise skin closure necessitating a free flap, then an option like a hindfoot nail should be used with a less complex wound closure.

The use of negative pressure dressing over a closed wound is a valuable adjunct in high-risk wounds, to support the skin closure and neutralize tension. Incisional negative pressure dressings over closed wounds have been found to decrease rates of wound dehiscence and wound infections in high-risk lower limb fractures.^22^ These finding have been corroborated in a meta-analysis demonstrating reduced superficial and deep wound infections.^23^

**Fig. 2:**
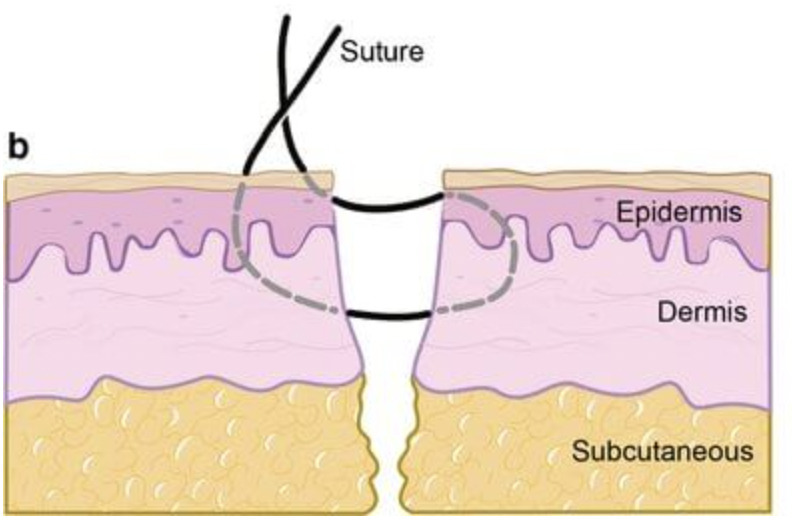
Image demonstrating the Allgöwer-Donati suture technique.

Another simple measure that can be used to optimize the condition of the soft-tissue envelope is the choice of suture technique. In both clinical and animal studies, the Allgöwer-Donati suture technique has been shown to decrease wound tension and have the lowest impact on capillary blood flow (Figure 2).^24,25^

### Use of single-stage surgery

This is perhaps the most controversial recommendation in this consensus opinion. Often the traditional management of open ankle fractures involves a primary debridement and skeletal stablization with temporary external fixation followed by definitive closure and internal fixation as a second stage. The principle of “span-scan-plan” that was originally developed for the management of pilon fractures is often widely applied to unstable ankle fractures.^26^ In an open ankle fracture, there are many of the same concerns with regards to soft-tissue compromise in a periarticular fracture. However, as highlighted previously, the low-energy open fragility fracture is a different clinical entity to the high-energy open tibial fracture. In the low-energy open ankle fracture, the wound is often a medial transverse split in which the skin has failed under tension (Figure 1). Furthermore, there is not the same degree of periosteal stripping and contamination.

Definitive fixation and soft-tissue closure or cover at the index operation has been shown to enable early mobilization, resulting in reduced length of stay and improved function.^27^ In a recent study of open ankle fractures, 56% of patients were managed with single-stage surgery, with limb salvage achieved in 93%.^8^ This strategy of single-stage surgery facilitated by a combined orthoplastic approach avoids prolonged bed rest, and eliminates the need for multiple anaesthetics in a vulnerable population. A single-stage approach will not be appropriate in every case. However, we challenge the routine practice of two-stage surgery in frail patients and promote definitive orthoplastic management in the index procedure wherever possible. This may result in different options for wound management and skeletal fixation being used, as explored in the next section.

### Unrestricted rehabilitation and early weightbearing

Unrestricted rehabilitation is facilitated by the use of surgical techniques that allow the patient to immediately fully weightbear. This minimizes the complications and morbidity of immobility in frail patients.^28^ There are many options available for fixation of an open ankle fracture, including joint preserving and joint sacrificing techniques. Irrespective of the surgeon’s preference for fixation method, the goal to fully weightbear must remain the same.

Joint-preserving methods with open reduction internal fixation (ORIF) can be intra- or extra-medullary, or external fixation. Joint-sacrificing methods include hindfoot nailing and fusion. Currently, there is no evidence to indicate a superior method of fixation. Achieving a congruent mortise and restoring alignment should be prioritized, rather than achieving an anatomical reduction of the articular surface.^29^

For the fibula fracture component, several options can be used, such as plate fixation or fibula nailing. Fibula nailing is growing in popularity, particularly in the presence of compromised tissues to provide a load bearing construct with rotational stability.^30^ Pro-tibial screws can be employed to enhance fixation to ensure a robust enough construct to endure full weightbearing.^31^ If a hindfoot nail or fusion strategy is selected, then the fibular does not require fixation.

Hindfoot nails, or tibiotalocalcaneal (TTC) nailing, has been shown to give good outcomes for the frail patient, with studies demonstrating up to 90% of patients who survived returned to their pre-hospital level of function.^7,32^ It has been demonstrated in a randomized controlled trial (RCT) that in closed, unstable fractures in frail patients, TTC nailing had a shorter length of stay and a lower complication rate compared to patients managed with ORIF.^33^ Further RCTs are underway to continue to investigate the superiority of TTC nails over ORIF.^34^

Many surgeons would choose a fine wire circular frame for the management of an open fracture. However, these have been associated with increased patient morbidity in frail patients, particularly if there is cognitive impairment.^20^ Definitive management with an external fixation device has also been associated with a significant increase in unplanned return to theatre.^35^

## Discussion

Guidelines exist for the management of ankle fractures, open fractures, and the frail patient. Frail patients with open ankle fractures are increasing in incidence and pose particular problems due to their bone quality, multiple comorbidities, and high complication rates. In addition, frail patients have increased risks associated with undergoing multiple surgeries, sarcopaenia, bed sores, hospital-acquired infections, and veno-thromboembolic events.

Consensus methods are being increasingly used to develop research agendas in various medical and surgical specialities, and we used this to help support establishing a consensus of appropriate management goals. Following a modified Delphi process, a mutually agreed consensus from the expert panel was reached to enable the best practice for the management of patients with frailty with an open ankle fracture: 1) All units managing lower limb fragility fractures should do so through a cohorted multidisciplinary pathway. This pathway should follow the standards laid down in the "care of the older or frail orthopaedic trauma patient" BOAST guideline.^16^ These patients have low bone density, and we should recommend full falls and bone health assessment. 2) All open lower limb fragility fractures should be treated in a single stage within 24 hours of injury if possible. 3) All patients with fragility fractures of the lower limb should be considered for mobilization on the day following surgery. 4) All patients with lower limb open fragility fractures should be considered for tissue sparing, judicious debridement as a default. 5) All patients with open lower limb fragility fractures should be managed by a consultant plastic surgeon with primary closure wherever possible. 6) The method of fixation must allow for immediate unrestricted weightbearing.

Consensus exercises, such as the Delphi process, have limitations; as the approach allows for the formulation of opinion by experts and equal contribution, it cannot replace rigorous scientific evidence. Indeed, there may be instances in which consensus does not reproduce the evidence, owing to misinformation or competing interests among the experts from whom opinion is sought.

In conclusion, by using experts from multiple specialities, the modified Delphi process has revealed agreement on the strategies required to manage open ankle fractures in patients with frailty. Importantly, a goal-directed approach that informs the clinical decision-making, operative planning, and rehabilitation to improve patient care and outcomes has been identified.

It is agreed several different techniques can be used to achieve these goals. Furthermore, this provides a basis to formulate and define best practices to ensure that the patient’s functional and rehabilitation requirements are at the forefront of all decision-making.

**Take home message**

- This consensus statement provides surgeons strategies to manage open ankle fractures in patients with frailty.

- Importantly, a goal-directed approach that informs the clinical decision-making, operative planning, and rehabilitation to improve patient care and outcomes has been identified.

## Data Availability

All data generated or analyzed during this study are included in the published article and/or in the supplementary material.
